# Crystal structure of (*E*)-4-hy­droxy-3-{1-[(4-hy­droxy­phen­yl)imino]­eth­yl}-6-methyl-2*H*-pyran-2-one

**DOI:** 10.1107/S2056989015012840

**Published:** 2015-07-11

**Authors:** Amel Djedouani, Sihem Boufas, Franck Cleymand, Michel François, Solenne Fleutot

**Affiliations:** a Laboratoire de Physicochimie Analytique et Cristallochimie de Matériaux, Organométalliques et Biomoléculaires, Université de Constantine 1, 25000 Constantine, Algeria; b Laboratoire de Génie Mécanique et Matériaux, Faculté de Technologie, Université 20 Aout 1955, 21000 Skikda, Algeria; c Institut Jean Lamour UMR 7198, Parc de Saurupt, CS 14234 F 54042 Nancy, France

**Keywords:** crystal structure, hy­droxy Schiff base, pyran-2-one, phenol–imine tautomer, hydrogen bonding, proton-transfer processes

## Abstract

In the title Schiff base, C_14_H_13_NO_4_, which adopts the phenol–imine tautomeric form, the dihedral angle between the planes of the benzene and heterocyclic (r.m.s. deviation = 0.037 Å) rings is 53.31 (11)°. An intra­molecular O—H⋯N hydrogen bond closes an *S*(6) ring. In the crystal, mol­ecules are linked by O—H⋯O hydrogen bonds to generate *C*(11) chains propagating in the [010] direction. A weak C—H⋯O link is also observed, leading to the formation of *R* ^5^_5_(32) rings extending parallel to the (101) plane.

## Related literature   

For photochromic and thermochromic properties of hy­droxy Schiff bases, see: Garnovskii *et al.* (1993 ▸); Hadjoudis *et al.* (2004 ▸). For potential materials for optical memory and switch devices, see: Zhao *et al.* (2007 ▸). For proton-transfer processes, see: Lussier *et al.* (1987 ▸). For Schiff base structures, see: Djedouani *et al.* (2007 ▸, 2008 ▸). For Schiff base bond lengths and angles, see: Girija & Begum (2004 ▸); Girija *et al.* (2004 ▸); Bai & Jing (2007 ▸).
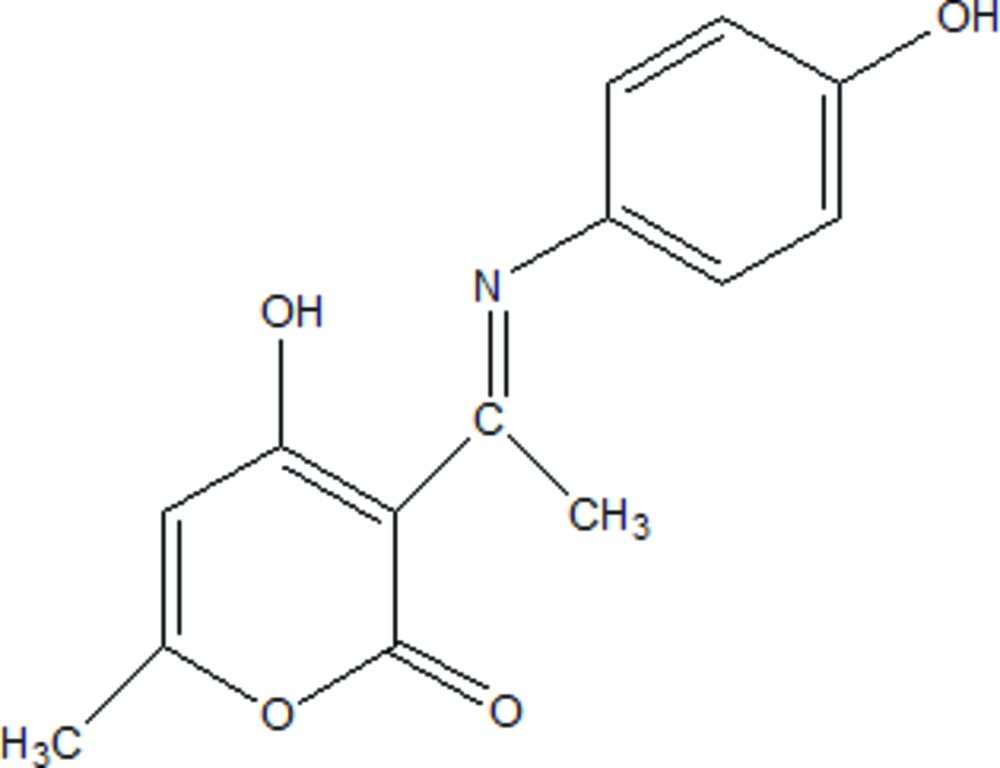


## Experimental   

### Crystal data  

C_14_H_13_NO_4_
*M* *_r_* = 259.26
Monoclinic, 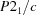

*a* = 7.8730 (5) Å
*b* = 11.7930 (8) Å
*c* = 13.5330 (8) Å
β = 99.896 (2)°
*V* = 1237.79 (14) Å^3^
*Z* = 4
Mo *K*α radiation
μ = 0.10 mm^−1^
*T* = 293 K
0.10 × 0.06 × 0.03 mm

### Data collection  

Nonius KappaCCD diffractometer
Absorption correction: multi-scan (*SADABS*; Sheldrick, 2002 ▸) *T*_min_ = 0.875, *T*_max_ = 0.947
17976 measured reflections
2582 independent reflections
2061 reflections with *I* > 2σ(*I*)
*R*_int_ = 0.026

### Refinement  

*R*[*F* ^2^ > 2σ(*F* ^2^)] = 0.047
*wR*(*F* ^2^) = 0.148
*S* = 1.07
2582 reflections
173 parameters
All H-atom parameters refined
Δρ_max_ = 0.54 e Å^−3^
Δρ_min_ = −0.38 e Å^−3^

### 

Data collection: *COLLECT* (Nonius, 2002 ▸); cell refinement: *DENZO-SMN* (Otwinowski & Minor, 1997 ▸); data reduction: *EVALCCD* (Duisenberg *et al.*, 2003 ▸); program(s) used to solve structure: *SIR97* (Altomare *et al.*, 1999 ▸); program(s) used to refine structure: *OLEX2.refine* (Dolomanov *et al.*, 2009 ▸); molecular graphics: *ORTEP-3 for Windows* (Farrugia, 2012 ▸) and *Mercury* (Macrae *et al.*, 2006 ▸); software used to prepare material for publication: *WinGX* (Farrugia, 2012 ▸) and *PARST* (Nardelli, 1995 ▸).

## Supplementary Material

Crystal structure: contains datablock(s) I. DOI: 10.1107/S2056989015012840/hb7460sup1.cif

Structure factors: contains datablock(s) I. DOI: 10.1107/S2056989015012840/hb7460Isup2.hkl

Supporting information file. DOI: 10.1107/S2056989015012840/hb7460Isup3.cml

**Fig. 1.**
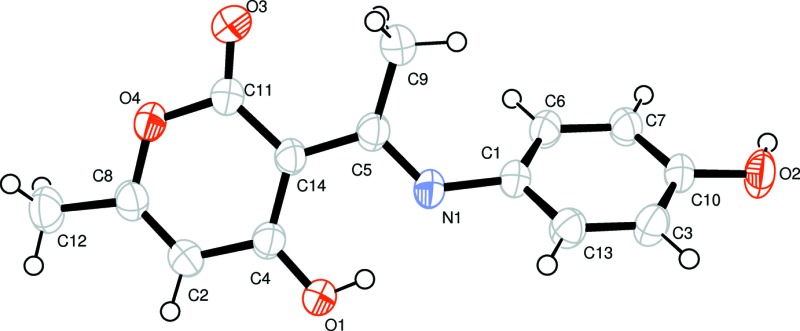
The structure of the title compound in 50% probability ellipsoids.

. DOI: 10.1107/S2056989015012840/hb7460fig1.tif
The structure of the title compound in 50% probability ellipsoids.

**Fig. 2.**
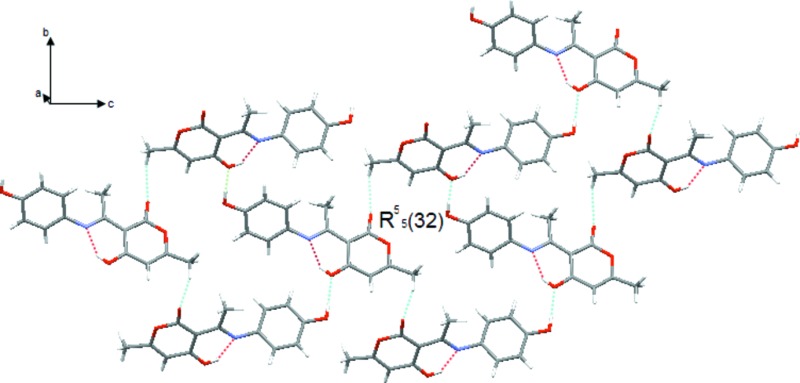
Part of the crystal structure of (I), showing the formation of S(6) rings with dashed red lines. C—H···O and O—H···O hydrogen bonds are shown as blue dashed lines.

S . DOI: 10.1107/S2056989015012840/hb7460fig2.tif
Part of the crystal structure of (I), showing the formation of *S*(6) rings with dashed red lines. N—H⋯O and O—H⋯O hydrogen bonds are shown as blue dashed lines.

CCDC reference: 1410367

Additional supporting information:  crystallographic information; 3D view; checkCIF report

## Figures and Tables

**Table 1:** Hydrogen-bond geometry (, )

*D*H*A*	*D*H	H*A*	*D* *A*	*D*H*A*
O1H1N1	0.82	1.83	2.560(2)	147
O2H2O1^i^	0.82	1.90	2.710(2)	169
C12H12BO3^ii^	0.96	2.55	3.137(3)	120

Symmetry codes: (i) 
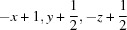
; (ii) 
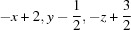
.

## supplementary crystallographic information

### S1. Comment

Hydroxy Schiff bases have been extensively studied due to their biological, photochromic and thermochromic properties (Garnovskii *et al.*, 1993; Hadjoudis *et al.*, 2004). They are potential materials for optical memory and switch devices (Zhao *et al.*, 2007). Proton transfer in these compounds forms the basis for an explanation of the mechanisms of various biological processes where proton transfer is the rate-determining step (Lussier *et al.*, 1987). In general, *O*-hydroxy Schiff bases exhibit two possible tautomeric forms, the phenol-imine (or benzenoid) and ketoamine (or quinoid) forms. Depending on the tautomers, two types of intra-molecular hydrogen bonds are possible: O—H···N in benzenoid and N—H···O in quinoid tautomers.

As part of our ongoing studies of Schiff bases (Djedouani *et al.*, 2007, 2008), we now describe the synthesis and the structure of the title compound, which takes the form phenol-imine and complete a six-membered pseudocycle *via* an intramolecular O—H····O hydrogen bond.

The dehydroacetic acid ring and phenyl ring are almost planer with r.m.s deviation for the mean plane are 0.0260 and 0.0027 respectively. The dihedral angle between the two rings is 53.30 (0.05) °. The two torsional angles τ1 (N1—C5—C14—C4) and τ2 (C5—N1—C1—C6) defining the confirmation of the molecule.

The N1—C5 distance of 1.324 (2) Å agree with similar bond in related compounds (Girija & Begum, 2004; Girija *et al.* 2004), slightly longer than a typical C=N bond (1.283 (4) Å) (Bai & Jing, 2007); but much shorter than the single carbon-nitrogen bond (Table. 1), N1—C1=1.432 (3) Å because of the resonance. The carbon-carbon bond connecting the phenol and imine groups exhibits intermediate distances between a single and a double bond and agree well with those observed in other azomethines. The C5—N1—C14 and C14—C5—N1 bond angle of 117.70 (2)° and 117.47 () ° respectively in the Schiff base ligand are smaller than typical hexagonal of 120°. This is due to the effect of substitution on O of pyron & OH of the DHA ring.

In the crystal, molecules are aligned head to foot along *b* axis, in columns along to [0 0 1] axis and the structure is stabilized by an O—H···O hydrogen bond and another weak C—H···O interaction, leading to the formation of *R*_5_^5^(32) rings extending parallel to the (101) plane (Fig. 2, Table.1).

### S2. Experimental

Compound (I) was prepared by refluxing a mixture of a solution containing dehydroacetic acid (0.01 mmol) and *para*-4-aminophenol (0.01 mmol) in ethanol (40 ml). The reaction mixture was stirred for 2 h under reflux and left to cool. Yellow crystals grew after a few days.

### S3. Refinement

C—H and O—H hydrogen atoms were placed in calculated positions and refined as riding atoms with C—H distances of 0.93 Å with *U*_iso_(H) = 1.2Ueq(C) and O—H distances of 0.82 Å, with *U*_iso_(H) = 1.2Ueq(N).

The methyl H atoms were constrained to an ideal geometry (C—H = 0.96 Å) with *U*_iso_(H) = 1.2Ueq(C), but were allowed to rotate freely about the C—C bonds.

### Crystal data

**Table:**

C_14_H_13_NO_4_	*F*(000) = 544.3271
*M**_r_* = 259.26	*D*_x_ = 1.391 Mg m^−^^3^
Monoclinic, *P*2_1_/*c*	Mo *K*α radiation, λ = 0.71073 Å
Hall symbol: -P 2ybc	Cell parameters from 0 reflections
*a* = 7.8730 (5) Å	θ = 2.9–27.5°
*b* = 11.7930 (8) Å	µ = 0.10 mm^−^^1^
*c* = 13.5330 (8) Å	*T* = 293 K
β = 99.896 (2)°	Block, yellow
*V* = 1237.79 (14) Å^3^	0.10 × 0.06 × 0.03 mm
*Z* = 4	

### Data collection

**Table:**

Nonius KappaCCD diffractometer	2582 independent reflections
Radiation source: Enraf–Nonius FR590	2061 reflections with *I* > 2σ(*I*)
Graphite monochromator	*R*_int_ = 0.026
Detector resolution: 9 pixels mm^-1^	θ_max_ = 26.7°, θ_min_ = 2.3°
CCD rotation images, thin slices scans	*h* = −9→9
Absorption correction: multi-scan (*SADABS*; Sheldrick, 2002)	*k* = −14→14
*T*_min_ = 0.875, *T*_max_ = 0.947	*l* = −17→17
17976 measured reflections	

### Refinement

**Table:**

Refinement on *F*^2^	Primary atom site location: structure-invariant direct methods
Least-squares matrix: full	Secondary atom site location: difference Fourier map
*R*[*F*^2^ > 2σ(*F*^2^)] = 0.047	Hydrogen site location: inferred from neighbouring sites
*wR*(*F*^2^) = 0.148	All H-atom parameters refined
*S* = 1.07	*w* = 1/[σ^2^(*F*_o_^2^) + (0.0655*P*)^2^ + 0.6848*P*] where *P* = (*F*_o_^2^ + 2*F*_c_^2^)/3
2582 reflections	(Δ/σ)_max_ = 0.005
173 parameters	Δρ_max_ = 0.54 e Å^−^^3^
0 restraints	Δρ_min_ = −0.37 e Å^−^^3^

### Fractional atomic coordinates and isotropic or equivalent isotropic displacement parameters (Å^2^)

**Table:**

	*x*	*y*	*z*	*U*_iso_*/*U*_eq_	
O1	0.6637 (2)	−0.08723 (12)	0.50633 (10)	0.0520 (4)	
H1	0.6696 (2)	−0.05535 (12)	0.45317 (10)	0.0779 (6)*	
O2	0.4993 (2)	0.21003 (13)	−0.00201 (10)	0.0543 (4)	
H2	0.4624 (2)	0.27519 (13)	−0.00450 (10)	0.0815 (7)*	
O3	1.0343 (3)	0.20593 (17)	0.65333 (13)	0.0814 (7)	
O4	0.92760 (19)	0.08216 (12)	0.74439 (9)	0.0443 (4)	
C1	0.6821 (3)	0.11371 (17)	0.29043 (13)	0.0390 (4)	
C2	0.7568 (3)	−0.07161 (18)	0.67874 (15)	0.0444 (5)	
H2a	0.7031 (3)	−0.13959 (18)	0.68968 (15)	0.0533 (6)*	
C3	0.6279 (3)	0.07382 (18)	0.11415 (14)	0.0433 (5)	
H3	0.6333 (3)	0.02421 (18)	0.06136 (14)	0.0520 (6)*	
C4	0.7488 (3)	−0.03156 (16)	0.57810 (14)	0.0391 (4)	
C5	0.8305 (3)	0.12191 (16)	0.46720 (14)	0.0382 (4)	
C6	0.6138 (3)	0.22135 (17)	0.27136 (14)	0.0416 (5)	
H6	0.6089 (3)	0.27092 (17)	0.32425 (14)	0.0499 (6)*	
C7	0.5531 (3)	0.25514 (17)	0.17428 (14)	0.0416 (5)	
H7	0.5074 (3)	0.32751 (17)	0.16191 (14)	0.0499 (6)*	
C8	0.8390 (3)	−0.01396 (17)	0.75665 (14)	0.0407 (5)	
C9	0.9259 (3)	0.22691 (19)	0.44950 (16)	0.0489 (5)	
H9a	0.9215 (18)	0.2372 (8)	0.37874 (17)	0.0733 (8)*	
H9b	0.8738 (13)	0.2909 (3)	0.4764 (11)	0.0733 (8)*	
H9c	1.0438 (6)	0.2203 (6)	0.4820 (10)	0.0733 (8)*	
C10	0.5598 (3)	0.18172 (17)	0.09476 (13)	0.0389 (4)	
C11	0.9398 (3)	0.12553 (18)	0.65006 (15)	0.0454 (5)	
C12	0.8492 (3)	−0.0416 (2)	0.86465 (15)	0.0554 (6)	
H12a	0.9678 (4)	−0.0434 (15)	0.8967 (3)	0.0831 (9)*	
H12b	0.789 (2)	0.0151 (9)	0.8959 (3)	0.0831 (9)*	
H12c	0.797 (2)	−0.1144 (7)	0.87109 (15)	0.0831 (9)*	
C13	0.6877 (3)	0.03998 (17)	0.21150 (15)	0.0419 (5)	
H13	0.7319 (3)	−0.03271 (17)	0.22414 (15)	0.0502 (6)*	
C14	0.8397 (2)	0.07122 (16)	0.56378 (13)	0.0368 (4)	
N1	0.7321 (2)	0.07168 (14)	0.39048 (12)	0.0434 (4)	

### Atomic displacement parameters (Å^2^)

**Table:**

	*U*^11^	*U*^22^	*U*^33^	*U*^12^	*U*^13^	*U*^23^
O1	0.0748 (11)	0.0408 (8)	0.0347 (7)	−0.0120 (7)	−0.0066 (7)	0.0022 (6)
O2	0.0822 (11)	0.0507 (9)	0.0265 (7)	0.0122 (8)	−0.0009 (7)	0.0018 (6)
O3	0.1131 (16)	0.0775 (13)	0.0444 (9)	−0.0536 (12)	−0.0125 (9)	0.0064 (9)
O4	0.0563 (9)	0.0434 (8)	0.0295 (7)	−0.0021 (6)	−0.0029 (6)	−0.0003 (6)
C1	0.0454 (11)	0.0404 (10)	0.0286 (9)	−0.0016 (8)	−0.0009 (7)	0.0027 (8)
C2	0.0565 (13)	0.0373 (10)	0.0383 (10)	−0.0024 (9)	0.0051 (9)	0.0042 (8)
C3	0.0508 (12)	0.0445 (11)	0.0329 (10)	0.0055 (9)	0.0021 (8)	−0.0065 (8)
C4	0.0466 (11)	0.0347 (10)	0.0332 (9)	0.0032 (8)	−0.0009 (8)	−0.0006 (8)
C5	0.0431 (10)	0.0367 (10)	0.0329 (9)	0.0046 (8)	0.0012 (8)	0.0004 (8)
C6	0.0557 (12)	0.0394 (10)	0.0290 (9)	0.0000 (9)	0.0052 (8)	−0.0029 (8)
C7	0.0532 (12)	0.0359 (10)	0.0343 (10)	0.0040 (9)	0.0036 (8)	0.0028 (8)
C8	0.0475 (11)	0.0389 (10)	0.0346 (10)	0.0077 (8)	0.0046 (8)	0.0027 (8)
C9	0.0536 (13)	0.0506 (12)	0.0397 (11)	−0.0060 (10)	0.0007 (9)	0.0079 (9)
C10	0.0451 (11)	0.0425 (11)	0.0275 (9)	0.0004 (8)	0.0018 (7)	0.0032 (8)
C11	0.0555 (13)	0.0423 (11)	0.0348 (10)	−0.0052 (10)	−0.0028 (9)	0.0032 (8)
C12	0.0773 (16)	0.0557 (14)	0.0329 (11)	0.0044 (12)	0.0088 (10)	0.0034 (9)
C13	0.0462 (11)	0.0379 (10)	0.0385 (10)	0.0066 (8)	−0.0008 (8)	0.0005 (8)
C14	0.0439 (11)	0.0336 (9)	0.0306 (9)	0.0029 (8)	−0.0002 (8)	0.0021 (7)
N1	0.0560 (10)	0.0414 (9)	0.0290 (8)	−0.0014 (8)	−0.0033 (7)	0.0036 (7)

### Geometric parameters (Å, º)

**Table:**

O1—H1	0.82	C5—C9	1.489 (3)
O1—C4	1.265 (2)	C5—C14	1.428 (3)
O2—H2	0.82	C5—N1	1.324 (2)
O2—C10	1.356 (2)	C6—H6	0.93
O3—C11	1.201 (3)	C6—C7	1.377 (3)
O4—C8	1.356 (2)	C7—H7	0.93
O4—C11	1.394 (2)	C7—C10	1.389 (3)
C1—C6	1.385 (3)	C8—C12	1.486 (3)
C1—C13	1.384 (3)	C9—H9a	0.96
C1—N1	1.432 (2)	C9—H9b	0.96
C2—H2a	0.93	C9—H9c	0.96
C2—C4	1.433 (3)	C11—C14	1.442 (3)
C2—C8	1.326 (3)	C12—H12a	0.96
C3—H3	0.93	C12—H12b	0.96
C3—C10	1.388 (3)	C12—H12c	0.96
C3—C13	1.380 (3)	C13—H13	0.93
C4—C14	1.438 (3)		
			
C4—O1—H1	109.5	C12—C8—C2	127.3 (2)
C10—O2—H2	109.5	H9a—C9—C5	109.5
C11—O4—C8	122.46 (15)	H9b—C9—C5	109.5
C13—C1—C6	119.66 (17)	H9b—C9—H9a	109.5
N1—C1—C6	121.88 (17)	H9c—C9—C5	109.5
N1—C1—C13	118.18 (18)	H9c—C9—H9a	109.5
C4—C2—H2a	119.25 (12)	H9c—C9—H9b	109.5
C8—C2—H2a	119.25 (12)	C3—C10—O2	117.93 (17)
C8—C2—C4	121.5 (2)	C7—C10—O2	122.75 (18)
C10—C3—H3	119.90 (11)	C7—C10—C3	119.31 (17)
C13—C3—H3	119.90 (12)	O4—C11—O3	113.30 (18)
C13—C3—C10	120.19 (18)	C14—C11—O3	129.00 (19)
C2—C4—O1	119.33 (18)	C14—C11—O4	117.69 (18)
C14—C4—O1	122.99 (17)	H12a—C12—C8	109.5
C14—C4—C2	117.68 (17)	H12b—C12—C8	109.5
C14—C5—C9	123.23 (17)	H12b—C12—H12a	109.5
N1—C5—C9	119.29 (17)	H12c—C12—C8	109.5
N1—C5—C14	117.48 (18)	H12c—C12—H12a	109.5
H6—C6—C1	119.91 (11)	H12c—C12—H12b	109.5
C7—C6—C1	120.17 (18)	C3—C13—C1	120.30 (18)
C7—C6—H6	119.91 (12)	H13—C13—C1	119.85 (11)
H7—C7—C6	119.82 (12)	H13—C13—C3	119.85 (12)
C10—C7—C6	120.36 (18)	C5—C14—C4	121.89 (17)
C10—C7—H7	119.82 (11)	C11—C14—C4	118.74 (17)
C2—C8—O4	121.50 (18)	C11—C14—C5	119.35 (18)
C12—C8—O4	111.21 (18)	C5—N1—C1	127.99 (17)

### Hydrogen-bond geometry (Å, º)

**Table:**

*D*—H···*A*	*D*—H	H···*A*	*D*···*A*	*D*—H···*A*
O1—H1···N1	0.82	1.83	2.560 (2)	147
O2—H2···O1^i^	0.82	1.90	2.710 (2)	169
C12—H12B···O3^ii^	0.96	2.55	3.137 (3)	120

Symmetry codes: (i) −*x*+1, *y*+1/2, −*z*+1/2; (ii) −*x*+2, *y*−1/2, −*z*+3/2.

## References

[bb1] Altomare, A., Burla, M. C., Camalli, M., Cascarano, G. L., Giacovazzo, C., Guagliardi, A., Moliterni, A. G. G., Polidori, G. & Spagna, R. (1999). *J. Appl. Cryst.* **32**, 115–119.

[bb2] Bai, Z.-C. & Jing, Z.-L. (2007). *Acta Cryst.* E**63**, o3822.

[bb3] Djedouani, A., Bendaas, A., Boufas, S., Allain, M., Bouet, G. & Khan, M. (2007). *Acta Cryst.* E**63**, o1271–o1273.

[bb4] Djedouani, A., Boufas, S., Allain, M., Bouet, G. & Khan, M. (2008). *Acta Cryst.* E**64**, o1785. 10.1107/S1600536808026032 PMC2960563 21201765

[bb5] Dolomanov, O. V., Bourhis, L. J., Gildea, R. J., Howard, J. A. K. & Puschmann, H. (2009). *J. Appl. Cryst.* **42**, 339–341.

[bb6] Duisenberg, A. J. M., Kroon-Batenburg, L. M. J. & Schreurs, A. M. M. (2003). *J. Appl. Cryst.* **36**, 220–229.

[bb7] Farrugia, L. J. (2012). *J. Appl. Cryst.* **45**, 849–854.

[bb8] Garnovskii, A. D., Nivorozhkin, A. L. & Minkin, V. I. (1993). *Coord. Chem. Rev.* **126**, 1–69.

[bb9] Girija, C. R. & Begum, N. S. (2004). *Acta Cryst.* E**60**, o535–o536.

[bb10] Girija, C. R., Begum, N. S., Sridhar, M. A., Lokanath, N. K. & Prasad, J. S. (2004). *Acta Cryst.* E**60**, o586–o588.

[bb11] Hadjoudis, E., Rontoyianni, A., Ambroziak, K., Dziembowska, T. & Mavridis, I. M. (2004). *J. Photochem. Photobiol. Chem.* **162**, 521–530.

[bb12] Lussier, L. S., Sandorfy, C., Le Thanh Hoa & Vocelle, D. (1987). *J. Phys. Chem.* **91**, 2282–2287.

[bb13] Macrae, C. F., Edgington, P. R., McCabe, P., Pidcock, E., Shields, G. P., Taylor, R., Towler, M. & van de Streek, J. (2006). *J. Appl. Cryst.* **39**, 453–457.

[bb14] Nardelli, M. (1995). *J. Appl. Cryst.* **28**, 659.

[bb15] Nonius (2002). *COLLECT*. Nonius BV, Delft, The Netherlands.

[bb16] Otwinowski, Z. & Minor, W. (1997). *Methods in Enzymology*, Vol. 276, *Macromolecular Crystallography*, Part A, edited by C. W. Carter Jr & R. M. Sweet, pp. 307–326. New York: Academic Press.

[bb17] Sheldrick, G. M. (2002). *SADABS*. Bruker AXS Inc., Madison, Wisconsin, USA.

[bb18] Zhao, L., Hou, Q., Sui, D., Wang, Y. & Jiang, S. (2007). *Spectrochim. Acta A Mol. Biomol. Spectrosc.* **67**, 1120–1125. 10.1016/j.saa.2006.09.033 17097913

