# Supplementary figures and images for: Crystal structure of (E)-4-hy­droxy-3-{1-[(4-hy­droxy­phen­yl)imino]­eth­yl}-6-methyl-2H-pyran-2-one
Source: Acta Crystallogr E Crystallogr Commun. 2015 Jul 11;71(Pt 8):o564–5. doi: 10.1107/S2056989015012840 (PMC4571398; doi:10.1107/S2056989015012840)

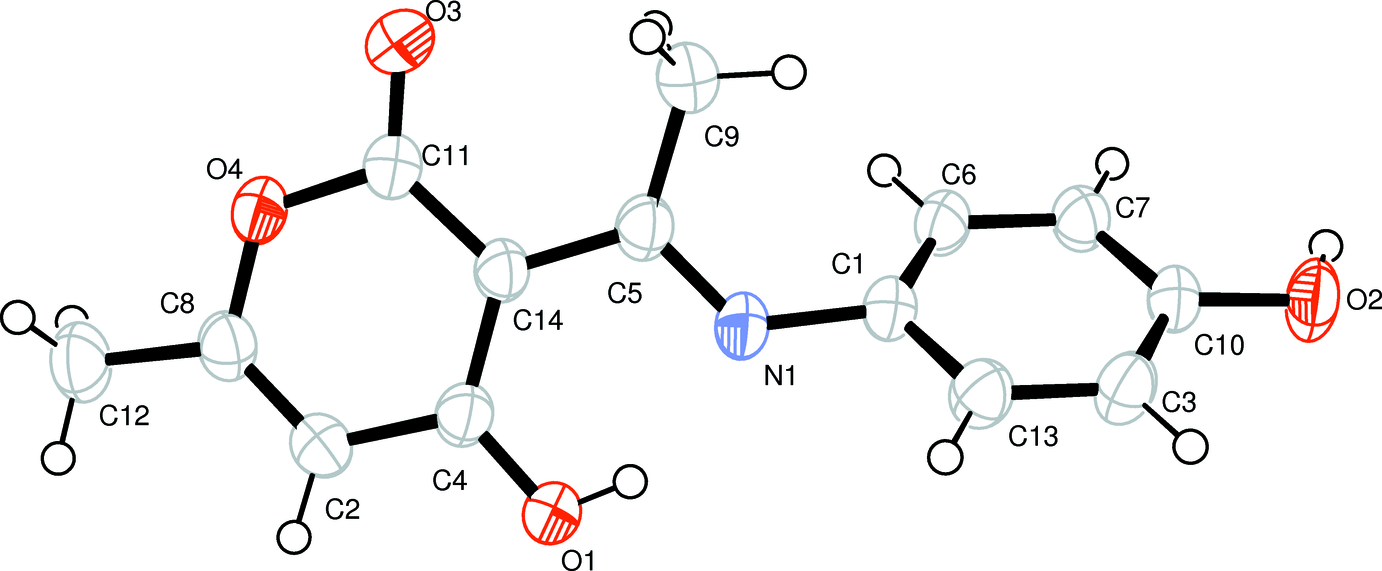

Supplement: Supplementary file 4 [file e-71-0o564-fig1.tif]

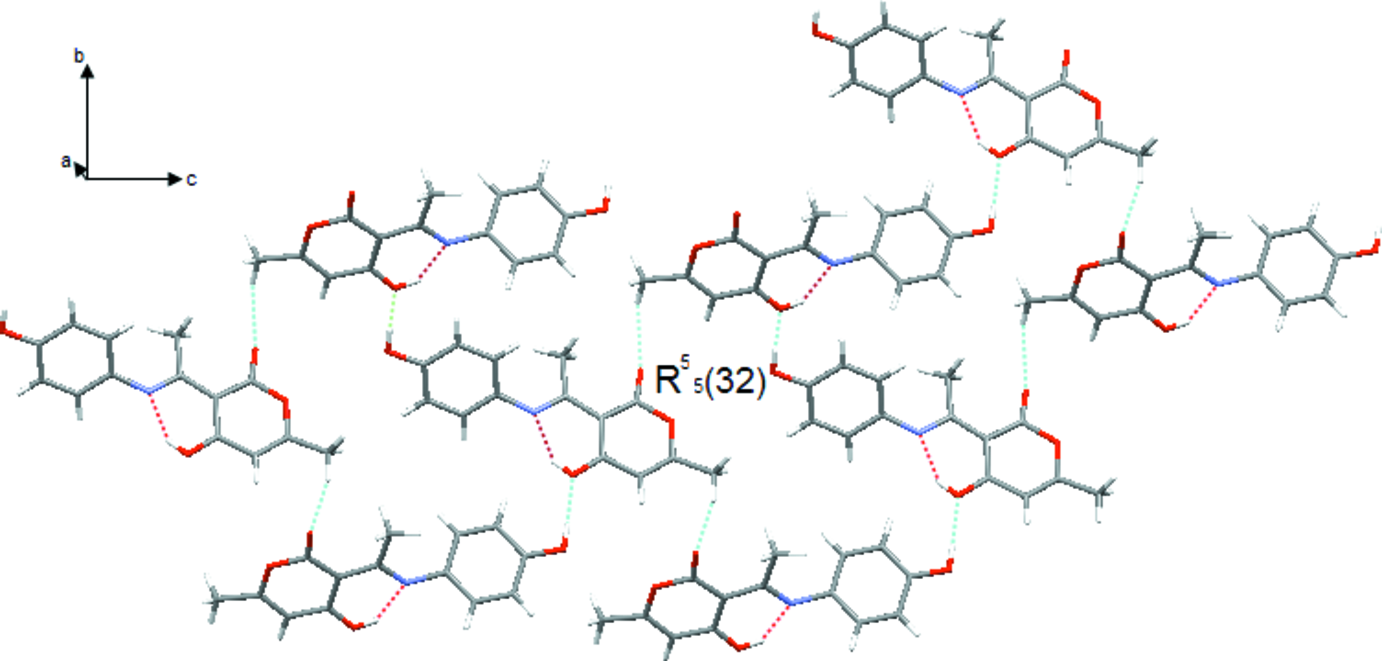

Supplement: Supplementary file 5 [file e-71-0o564-fig2.tif]
